# Dynamic postural stability in individuals with ACL reconstruction versus healthy controls with insights into sex differences: A cross-sectional study

**DOI:** 10.1371/journal.pone.0351496

**Published:** 2026-06-12

**Authors:** Wasim Labban, Juan Forero, Lindsey Westover, Mark Sommerfeldt, Stephanie Nathanail, Lauren Beaupre

**Affiliations:** 1 Faculty of Rehabilitation Medicine, University of Alberta, Edmonton, Canada,‌‌; 2 Mirdif Center for Physiotherapy and Rehabilitation, Dubai, United Arab Emirates; 3 Human Movement Laboratory, Faculty of Rehabilitation Medicine, University of Alberta, Edmonton, Canada; 4 Faculty of Engineering, University of Alberta, Edmonton, Canada; 5 Division of Orthopedic Surgery, Department of Surgery, Faculty of Medicine & Dentistry, University of Alberta, Edmonton, Canada; 6 Glen Sather Sports Medicine Clinic, University of Alberta, Edmonton, Canada; 7 Clinical Trial Unit, Alberta Health Services, Edmonton, Alberta, Canada; Juntendo University, JAPAN

## Abstract

**Objectives:**

To compare dynamic postural stability, measured by time to stabilization (TTS) and postural stability indices (PSI), after double-leg counter-movement jump (CMJ) landing in individuals 9–24 months following anterior cruciate ligament reconstruction (ACLR) and healthy controls. Additionally, to explore the effect of sex and ACLR status on postural stability.

**Methods:**

This cross-sectional laboratory-based study included 41 participants: 21 individuals (10 females) 9–24 months post-ACLR and 20 healthy controls (10 females). Participants performed double-leg countermovement jumps (CMJs) on force plates, landed, and maintained the landing position for 10 seconds. Time to stabilization (TTS), defined as the time (s) required for the ground reaction force to reach and maintain a stable state following landing, and postural stability index (PSI), a composite measure of the ability to maintain equilibrium during the transition from dynamic to static conditions, were calculated and compared between groups.

**Results:**

The ACLR group exhibited significantly higher TTS values than healthy controls, indicating a longer duration to achieve stability. Specifically, the resultant vector TTS when combined from both force plates (RVTTS-C), and the vertical TTS in the operated leg (VTTS-op) was higher in the ACLR than the healthy controls (p = 0.03, p = 0.02, respectively). Furthermore, males with ACLR demonstrated higher VTTS combined (VTTS-C) and VTTS-op than females post-ACLR (p = 0.03, p < 0.01, respectively) and higher VTTS-op compared to healthy males (p = 0.03). There were no differences in PSIs between groups.

**Conclusion:**

Our study revealed significant deficits in dynamic postural stability in individuals post ACLR, with notable sex differences. The findings suggest a need for targeted neuromuscular rehabilitation to improve landing stability post ACLR and reduce the risk of secondary injury. Further research is needed to understand sex-specific postural stability mechanisms for tailored rehabilitation.

## Introduction

Anterior cruciate ligament (ACL) rupture is a devastating injury with a high incidence that continue to rise globally [1]. The estimated global incidence of ACL injuries is approximately 68.6 per 100,000 person-year [2]. Most of ACL injuries occur without a direct knee contact [3] when the limb decelerates during landing or change of direction activities such as cutting and pivoting [4]. Due to the vital role of the ACL in the knee joint stabilization [5], ACL injuries can lead to impairments of the knee stability and balance [6]. Therefore, anterior cruciate ligament reconstruction (ACLR) is frequently indicated to restore knee joint stability. ACLR is usually followed by a course of rehabilitation with the ultimate aim of returning patients to their pre-injury level of function.

Beyond the surgical technique, the success of ACL reconstruction hinges on the effectiveness of the post-operative rehabilitation protocol [7]. Traditional protocols often focus on strength and range of motion; however, evidence supports accelerated programs that incorporate early weight-bearing and intensive neuromuscular training [8]. Furthermore, the clinical use of functional or proprioceptive knee bracing can complement these rehabilitative efforts by enhancing proprioception and reducing kinesiophobia in patients during the transition back to high-demand activities [9]**.** Specifically, neuromuscular and perturbation training are critical for re-establishing sensorimotor control and correcting abnormal postural strategies that often persist for years after surgery [10].

Dynamic postural stability is a critical aspect of movement control and injury prevention, particularly after ACLR [11]. Individuals often face challenges in regaining optimal postural stability, which can significantly impact their functional performance and risk of re-injury [12]. Understanding the nuances of postural stability in individuals post-ACLR compared to healthy controls is essential for tailoring rehabilitation programs and enhancing long-term outcomes [13].

In a clinical setting, postural stability is usually tested statically. Patients are asked to stand on a single leg while maintaining their balance on a stationary platform. Several parameters related to the center of pressure can then be compared between the injured and non-injured legs or between patients and healthy controls [14]. Although static balance testing may be valuable for measuring postural control [15], static postural stability measures have been described as non-functional, insensitive, and do not correlate with dynamic values [16]. Therefore, measurement of more challenging and dynamic aspects of postural control that mimic athletic activities may provide important insight into athletes’ functional abilities with and without ACLR [15,17].

Assessing postural stability after performing a jump task, such as the countermovement jump (CMJ), provides valuable insights into lower limb function and dynamic stability. While previous studies have predominantly focused on single-leg CMJ tasks, examining postural stability immediately after double-leg CMJ landing offers a comprehensive evaluation of bilateral weight-bearing capabilities and control mechanisms of both lower limbs [18]. Various methods exist to calculate and report parameters to estimate postural stability, each with its own advantages and limitations [19].

Time to stabilization (TTS) and the dynamic postural stability index (DPSI) have been suggested as metrics for evaluating postural stability [19,20]. They both reflect an individual’s capacity to maintain stability during the shift from a dynamic to a static state [21,22]. TTS is the duration required for the ground reaction force (GRF) signal to stabilize following a landing on a force plate [20] while DPSI quantifies deviations around a central point to assess fluctuations in stability [19].

Several studies have also observed sex differences after ACLR in various measures of physical function, for example, hop performance [23], patients reported outcome measures (PROMs) [24,25], and 3D knee kinematics [23,26]. While the impact of sex on postural stability showed mixed findings with different populations according to a systematic review [27], there is an inconclusive evidence regarding the influence of sex on measures of postural stability in individuals following ACLR.

Our recent systematic review provided a comprehensive overview of several force plate parameters and their discriminative abilities in individuals post-ACLR during CMJs and drop jumps [28]. However, none of the identified outcomes addressed postural stability during double-leg CMJ landing in individuals with and without ACLR [28]. Therefore, the primary of objective of this study was to compare dynamic postural stability immediately after double-leg CMJ landing in individuals between 9- and 24-months following ACLR and healthy controls. Additionally, an exploratory objective was to assess the potential association between sex and ACLR status in relation to postural stability outcomes. We hypothesized that individuals post-ACLR would exhibit impaired dynamic postural stability, characterized by prolonged TTS and increased PSI values compared to healthy controls. Furthermore, we hypothesized that sex-specific differences in stabilization performance would be observed.

## Materials and methods

### Study design

This cross-sectional study was conducted and reported using the Strengthening the Reporting of Observational studies in Epidemiology (STROBE) guidelines [29] and the considerations for methodological reporting suggested in our previous work [30]. The study protocol was approved by the Research Ethics Board at the University of Alberta (Pro00111475).

### Setting

Testing procedures were performed at the Human Movement Laboratory at the University of Alberta, Edmonton, Alberta, Canada. We recruited a convenience sample of participants with and without ACLR. Participants were recruited through study posters displayed at the University of Alberta and local community sport centres. A digital copy of the study poster was also distributed to students in the Faculty of Rehabilitation Medicine and the Department of Mechanical Engineering at the University of Alberta. Most participants with ACLR were recruited from the Glen Sather Sports Medicine Clinic, University of Alberta. Participants recruitment took a place between December 9, 2022, and May 2, 2024. In addition, data from 10 participants who were 9–12 months post-ACLR were included from an ongoing cohort study that used the same inclusion/exclusion criteria, testing protocol, equipment, and laboratory setting.

### Inclusion and exclusion criteria

Participants in the ACLR group were recruited if they had undergone primary unilateral ACLR within the past 9–24 months, reported no significant concomitant ligamentous injuries, had no bilateral lower limb pathologies or recent orthopedic surgeries, reported regular participation in sports/activities involving jumping, cutting, pivoting, and lateral movements prior to the ACL injury, and were planning to return to their sports/activities or had already done so, and had initiated dynamic activities during their ACL rehabilitation. Participants in the healthy control group were recruited if they were participating in sports/activities involving jumping, cutting, pivoting, and lateral movements and had no history of knee injuries requiring medical intervention. Participants in both groups were between 18 and 35 years old and had no health conditions affecting participation.

Participants were excluded if they had back pain, recent concussion, a balance disorder, scored less than 80% on the knee extensors isokinetic strength symmetry index, or if they were found not fit for jumping/landing activities according to knee clinical examinations performed by a qualified research staff. All participants signed an informed consent form approved by the Research Ethics Board.

### Instrumentation

Ground reaction force (GRF) data were collected separately for each leg by means of two 46.5 cm wide × 51 cm long force plates (model AMTI OR6-7-1, AMTI, Watertown, MA, USA) located side-by-side with no gap between them and mounted in the center of a custom-built 122 cm wide x 173 cm long platform. Data for the GRF (sampled at 1000 Hz) were recorded and processed using the Motion Monitor xGen v3.80.3.0 system (Innovative Sports Training, Inc, Chicago, IL, USA) to output the vertical (GRFv), medial-lateral (GRFml) and anterior-posterior (GRFap) force component traces from 3 seconds before the jump and 15 seconds after the landing.

The bioelectrical impedance analysis (BIA) device (Tanita MC-780U) was used to estimate overall body composition. The knee isokinetic dynamometry testing was done using the PrimusRS system (BTE PrimusRS (PR30); BTE Technologies, Hanover, MD, USA).

### Procedure

Participants underwent a standardized laboratory procedure to assess various aspects of knee function (isokinetic strength testing) and postural stability (force plate analysis). Anthropometric measurements, including height, weight, and bilateral thigh circumference, were taken. Participants also stood on a BIA to estimate overall body composition. A standardized warm-up routine was conducted, comprising stationary cycling, dynamic lower body mobility exercises, stretching, and submaximal body-weight exercises. Subsequently, isokinetic dynamometry was performed to assess concentric knee flexion and extension strength. Participants were seated with the hip at approximately 90° of flexion. Stabilization was achieved using straps across the pelvis and both thighs to minimize compensatory movement. The dynamometer axis of rotation was aligned with the lateral femoral epicondyle. The resistance pad was positioned approximately 5 cm proximal to the lateral malleolus, with placement adjusted for flexion and extension testing. The range of motion was set from 0° (full extension) to 100° of knee flexion, verified using a goniometer. Testing was conducted at an angular velocity of 60°/s. Participants completed 3 familiarization trials and 5 maximal voluntary contractions of concentric knee flexion and extension per limb.

The knee extensors isokinetic strength symmetry index was calculated using the following formula:


$$\begin{array}{c} \frac{The\;average\;of\;5\;maximal\;contractions\;of\;knee\;extensors\;in\;the\;involved\;or\;non-dominant\;limb}{The\;average\;of\;5\;maximal\;contractions\;of\;knee\;extensors\;in\;the\;contralateral\;limb}x100 \end{array}$$

Participants who scored 80% or more on the isokinetic strength symmetry index moved on to the jump testing [31]. Participants who scored less than 80% were allowed another assessment within one month to re-test their strength stability indices to allow them to participate in the evaluation. If they were unsuccessful on the second attempt, they were excluded from the study.

### Jump testing

Participants were asked to perform a barefoot jump-specific warm-up protocol including 10 CMJs, 10–15 calf hops, and 10 CMJ rebound jumps. Participants were then asked to stand barefoot with each foot on a force plate, positioned shoulder-width apart. They were asked to stand tall placing hands on hips maintaining this position over the respective force plate motionless for at least three seconds before jumping.

At the time of hearing the word “jump“, participants would rapidly perform a CMJ, which involves downward motion/squat to approximately 90 degrees of knee flexion, standardized through practice trials and visual feedback provided by the examiner to ensure consistent squat depth across participants, followed by rapid triple extension of the ankles, knees, and hips, jumping as high as possible, while maintaining hands on hips and landing with each foot completely on the respective force plate. Participants were required to maintain their landing position, without any movement, for a duration of 10 seconds after landing.

Participants were instructed to perform 5 CMJ trials, taking a minimum break of five seconds between each jump. Participants would repeat trials if jumps failed to meet the established criteria, such as any movement occurring before 10 seconds post-landing, incorrect execution of the jump, or landing with part of a foot off the force plates. To count for learning effect and fatigue, data from the first and last trials were excluded from data analysis [32].

### Outcomes

We assessed time to stabilization (TTS) and postural stability indices (PSIs) using four distinct measures for each outcome. For TTS, we evaluated anterior-posterior time to stabilization (APTTS), medial-lateral time to stabilization (MLTTS), vertical time to stabilization (VTTS), and resultant vector time to stabilization (RVTTS), which assesses TTS in both horizontal planes [33]. Similarly, for PSIs, we examined anterior-posterior stability index (APSI), medial-lateral stability index (MLSI), vertical stability index (VSI), and dynamic postural stability index (DPSI).

We compared the combined TTS (TTS-C) and combined PSIs (PSI-C) measured from both force plates (i.e., both limbs) between the ACLR and control groups. TTS-C and PSI-C were calculated from the combined forces (FC) from the forces in the right (FR) and left (FL) force plates using the following formula: FC = FR + FL. Additionally, we compared these variables from the operated leg in the ACLR group with the corresponding (right or left) sides in the control group, and repeated the comparison for the contralateral sides. Furthermore, we assessed the asymmetry in TTS and PSIs between the two legs in the ACLR group and compared it with the asymmetry observed in the control group. The full list of TTS and PSI outcomes with their abbreviation are listed in Table 1.

**Table 1:** List of TTS and PSI outcomes.

Time to stabilization		Postural stability indices	
Abbreviations	Outcomes	Abbreviations	Outcomes
APTTS-C	Anterior posterior time to stabilization-combined	**APSI-C**	Anterior posterior postural stability index – combined
MLTTS-C	Medial lateral time to stabilization-combined	**MLSI-C**	Medial lateral postural stability index – combined
VTTS-C	Vertical time to stabilization-combined	**VSI-C**	Vertical postural stability index – combined
RVTTS-C	Resultant vector time to stabilization-combined	**DPSI-C**	Dynamic postural stability index – combined
APTTS-op	Anterior posterior time to stabilization in operated leg	**APSI-op**	Anterior posterior postural stability index in operated leg
MLTTS-op	Medial lateral time to stabilization in operated leg	**MLSI-op**	Medial lateral postural stability index in operated leg
VTTS-op	Vertical time to stabilization in operated leg	**VSI-op**	Vertical postural stability index in operated leg
RVTTS-op	Resultant vector time to stabilization in operated leg	**DPSI-op**	Dynamic postural stability index in operated leg
APTTS-nop	Anterior posterior time to stabilization in non-operated leg	**APSI-nop**	Anterior posterior postural stability index in non-operated leg
MLTTS-nop	Medial lateral time to stabilization in non-operated leg	**MLSI-nop**	Medial lateral postural stability index in non-operated leg
VTTS-nop	Vertical time to stabilization in non-operated leg	**VSI-nop**	Vertical postural stability index in non-operated leg
RVTTS-nop	Resultant vector time to stabilization in non-operated leg	**DPSI-nop**	Dynamic postural stability index in non-operated leg
APTTS-Asy	Asymmetry in anterior posterior time to stabilization	**APSI-Asy**	Asymmetry in anterior posterior postural stability index
MLTTS-Asy	Asymmetry in medial lateral time to stabilization	**MLSI-Asy**	Asymmetry in medial lateral postural stability index
VTTS-Asy	Asymmetry in vertical time to stabilization	**VSI-Asy**	Asymmetry in vertical postural stability index
RVTTS-Asy	Asymmetry in resultant vector time to stabilization	**DPSI-Asy**	Asymmetry in dynamic postural stability index

**Note:** Time to stabilization (TTS) variables are reported in seconds. Postural stability indices (PSIs) are dimensionless. Asymmetry variables represent relative differences between limbs and are unitless.

### Data processing

A moving average filter with a 100-millisecond window was applied to the GRF traces to reduce random noise [34]; then a subset of data trimmed from the time of landing and lasting 10 seconds was extracted for the calculations. Time of landing was determined from the filtered traces as the time after jumping when GRFv > 10 Newtons, and body weight was estimated as the media value over the last 2 seconds of the data subset. GRF data from each force plate was used to analyze the stability performance from each side (TTS-left, TTS-right, PSIs-left, PSIs-right), and combined data from both force plates was used to analyze the participant’s overall stability (TTS-C, DPSI-C). Data from all the trials was processed offline using a custom-written program in R (R Core Team, 2023) [35] to calculate TTS variables [anterior-posterior TTS (APTTS), medio-lateral TTS (MLTTS), vertical TTS (VTTS)] and PSIs variables [anterior-posterior PSI (APSI), medio-lateral PSI (MLSI), vertical PSI (VSI), and dynamic PSI (DPSI)] based on the methods described by Colby et al. 1999 [20] and Wikstrom et al., 2005 [19], respectively. Specifically, TTS was defined as the time required for the GRF signals to return and remain within a stable range following landing, relative to zero in anterior-posterior and medio-lateral directions and relative to body weight in the vertical direction [20]. The resultant vector TTS (RVTTS) was calculated using the following formula $RVTTS=\sqrt{{\left(APTTS\right)}^{\text{2}}+{\left(MLTTS\right)}^{\text{2}}}$ [36]. PSIs were calculated as the root mean square of the deviations in GRFs from the references values over the analysis period [19].

### Statistical analysis

Sample size estimation was based on detecting differences in TTS between individuals post-ACLR and healthy controls, using data from previous literature looking at TTS after forward jump landing in females [21]. Using the Satterthwaite’s t-test assuming unequal variances, 20 participants per group would acquire a power of 82%. Analyses involving sex-specific subgroups were considered exploratory and were not specifically powered. All outcome variables were assessed for normality and homogeneity of variances using The Shapiro-Wilk’s test and the Levene’s tests, respectively.

As most of the TTS and PSI variables were not normally distributed, we applied the Mann-Whitney U test for all TTS and PSI comparisons regardless of their distribution to provide homogeneity of data reporting for these variables. Therefore, we used the median and the interquartile range (IQR) to examine potential differences between the ACLR and healthy control group, for all the TTS and PSI outcomes. For the participant characteristics, we applied parametric or non-parametric tests as appropriate based on data distribution. Significance was set at p < 0.05.

When significant differences were identified, we estimated effect sizes using Cliff’s Delta with the corresponding 95% confidence intervals. Cliff’s Delta was interpreted as trivial (δ < 0.11), small (0.11 ≤ δ < 0.28), medium (0.28 ≤ δ < 0.43), and large (δ ≥ 0.43) [37] Cliff’s Delta effect sizes were calculated using an Excel sheet (website: Real Statistics Using Excel, 2023) [38], while the rest of the statistical analysis were performed using Stata (StataCorp LLC, version 15.1, USA).

To explore the effect of sex and the ACLR status, we employed the Kruskal Wallis test. The Dunn’s post hoc test with Bonferroni adjustment was used for pair-wise comparisons, followed by effect size estimation for the significant comparisons. The dataset supporting the findings of this study is provided in S1 File.

## Results

A total of 43 participants were recruited, comprising 23 individuals with ACLR and 20 healthy controls. Among the ACLR group, two participants did not meet the criterion of 80% on the quadriceps strength symmetry index at either their initial or secondary assessment. Consequently, our final dataset comprised 41 participants, including 20 healthy individuals (10 females and 10 males) and 21 individuals in the ACLR group (10 females and 11 males). The participants’ characteristics are detailed in Table 2. Individuals in both groups exhibited similarities across numerous characteristics; however, Healthy controls consistently outperformed individuals with ACLR across various functional measures such as Tegner (p = 0.002), The Marx Activity Rating Scale (MARS) (p = 0.03), and the International Knee Documentation Committee (IKDC) (p < 0.001).

**Table 2:** Participants Characteristics.

Participants’ Characteristics	ACLR (n = 21) Mean ± SD			Healthy (n = 20) Mean ± SD			P-value
	Female (n = 10)	Male (n = 11)	Total (n = 21)	Female (n = 10)	Male (n = 10)	Total (n = 20)	
Age (year)†	20.25 (3.08)	28.50 (8.5)	22.17 (8.5)	22.63 (3.09)	22.21 (4.08)	22.63 (3.54)	0.97
Height (cm)	163.08 ± 6.05	175.98 ± 7.54	170.18 ± 9.15	161.6 ± 4.11	176.1 ± 12.73	168.85 ± 11.84	0.69
Weight (kg)	66.99 ± 12.06	82.89 ± 8.85	75.32 ± 13.07	61.95 ± 5.26	78.80 ± 10.18	70.38 ± 11.70	0.21
Total muscle mass (kg)	44.70 (8.10)	62.30 (9.80)	53.8 (16.9)	44.05 (2.00)	63.90 (13.50)	48.7 (19.85)	0.74
Trunk muscle mass (kg)†	26.20 (4.30)	31.60 (6.10)	29.3 (5.4)	25.55 (1.00)	33.5 (7.90)	28.4 (8.05)	0.97
Right leg muscle mass (kg)†	7.20 (1.40)	11.10 (1.20)	9.5 (3.6)	7.3 (0.80)	11.25 (1.8)	9.0 (4.2)	0.91
Left leg muscle mass (kg)†	7.10 (1.40)	11.00 (1.20)	9.3 (3.7)	7.15 (0.80)	8.9 (4.15)	8.9 (4.15)	0.93
Total fat mass (kg)	18.77 ± 7.32	17.22 ± 6.17	17.96 ± 6.62	15.83 ± 4.39	14.86 ± 4.39	14.86 ± 4.39	0.09
Total fat %	26.91 ± 5.91	20.55 ± 6.04	23.58 ± 6.68	22.85 ± 5.46	19.59 ± 6.11	19.59 ± 6.11	0.053
Trunk fat %	21.23 ± 7.13	22.82 ± 6.30	22.06 ± 6.53	17.29 ± 5.29	17.79 ± 5.41	17.79 ± 5.41	0.03*
Right leg fat %	34.11 ± 4.34	17.68 ± 7.05	25.43 ± 10.11	32.15 ± 3.59	22.86 ± 10.37	22.86 ± 10.37	0.43
Left leg fat %	25.62 ± 9.90	17.90 ± 6.42	25.62 ± 9.90	32.60 ± 3.59	23.28 ± 10.32	23.28 ± 10.32	0.49
Total body water %†	53.05 (6.80)	56.6 (8.4)	55.4 (7.2)	54.5 (5.10)	61.65 (7.20)	56.85 (8.35)	0.14
Tegner†	5 (3)	5 (1)	5 (1)	7 (3)	8 (2)	7 (3)	0.002*
MARS	9.8 ± 3.61	5.27 ± 4.20	7.43 ± 4.48	11.10 ± 3.45	9.60 ± 4.35	10.35 ± 3.90	0.032*
IKDC%†	85.05 (14.90)	86.2 (9.20)	86.2 (10.3)	99.45 (3.40)	99.45 (1.10)	99.5 (2.9)	<0.001*
Quadriceps strength SI%†	93 (7)	89 (7)	91 (12)	91.5 (11)	94 (12)	93 (11.5)	0.39
ACL-RSI	75.30 ± 30.17	64.45 ± 30.12	69.62 ± 29.90	NA	NA	NA	
TSK-11	17.80 ± 4.34	17.27 ± 5.71	17.52 ± 4.99	NA	NA	NA	
Time since surgery (month)†	10.82 (2.96)	9.80 (4.44)	10.07 (3.16)	NA	NA	NA	
Hamstring autograft, n (%)	8/10 (80)	11/11 (100)	19/21 (90.48)	NA	NA	NA	
Quadriceps autograft, n (%)	2/10 (20)	0/11 (0)	2/21 (9.52)	NA	NA	NA	
Co-injuries, n (%)	5/10 (50)	8/11 (72.73)	12/21 (57.14)	NA	NA	NA	
Meniscus, n (%)	4/10 (40)	6/11 (54.55)	10/21 (47.62)	NA	NA	NA	
Meniscus & chondral, n (%)	0/10 (0)	1/11 (9.09)	1 (4.76)	NA	NA	NA	
Meniscus & MCL sprain, n (%)	1/10 (10)	0/11 (0)	1 (4.76)	NA	NA	NA	

^†^Data reported in median and inter-quartile range (IQR), MARS: Marx Activity Rating Scale, IKDC: International Knee Documentation Committee, SI: symmetry index, ACLR-RSI: Anterior Cruciate Ligament-Return to Sport after Injury, TSK: Tempa Scale of Kinesiophobia, MCL: Medial Collateral Ligament.

### Time to stabilization

When comparing the ACLR group to the control group, significant differences were observed in the RVTTS-C and VTTS-op, with p-values of 0.03 and 0.02, respectively. Effect sizes were moderate for RVTTS-C (δ = 0.4, 95% CI [0.04, 0.67]) and large for VTTS-op (δ = 0.96, 95% CI [0.84, 0.99]). No other significant differences were found between the groups (see Table 3).

**Table 3:** Comparisons of TTS variables among ACLR and Healthy groups.

OUTCOMES	ACLR (n = 21)	Healthy (n = 20)	P-value	ES (95% CI)
APTTS-C	2.01 (0.28)	2.04 (0.23)	0.80	
MLTTS-C	2.29 (0.27)	2.16 (0.42)	0.38	
VTTS-C	1.61 (0.16)	1.57 (0.1)	0.07	
RVTTS-C	2.31 (1.02)	2.03 (0.26)	0.03*	**0.40 (0.04 - 0.67)**
APTTS-op	1.97 (0.29)	1.87 (0.27)	0.28	
MLTTS-op	1.93 (0.39)	1.83 (0.47)	0.30	
VTTS-op	1.76 (0.27)	1.6 (0.19)	0.02*	**0.96 (0.84 - 0.99)**
RVTTS-op	2.87 (0.35)	2.87 (0.35)	1.00	
APTTS-nop	2.12 (0.24)	2.19 (0.46)	0.56	
MLTTS-nop	1.95 (0.4)	1.84 (0.4)	0.16	
VTTS-nop	1.66 (0.18)	1.63 (0.16)	0.53	
RVTTS-nop	2.97 (0.47)	2.94 (0.56)	0.47	
APTTS-Asy	−0.01 (0.20)	0.01 (0.27)	0.71	
MLTTS-Asy	0.06 (0.23)	0.02 (0.19)	0.80	
VTTS-Asy	0.03 (0.27)	0.07 (0.20)	0.90	
RVTTS-Asy	0.02 (0.35)	0.01 (0.18)	0.72	

Data reported in median and inter-quartile range (IQR). APTTS-C: anterior-posterior time to stabilization combined from both force plates. MLTTS-C: medial-lateral time to stabilization combined from both force plates. VTTS-C: vertical time to stabilization combined from both force plates. RVTTS-C: resultant vector time to stabilization combined from both force plates. APTTS-op: anterior-posterior time to stabilization on the operated leg. MLTTS-op: medial-lateral time to stabilization on the operated leg. VTTS-op: vertical time to stabilization on the operated leg. RVTTS-op: resultant vector time to stabilization on the operated leg. APTTS-nop: anterior-posterior time to stabilization on the non-operated leg. MLTTS-nop: medial-lateral time to stabilization on the non-operated leg. VTTS-nop: vertical time to stabilization on the non-operated leg. RVTTS-nop: resultant vector time to stabilization on the non-operated leg. APTTS-Asy: asymmetry in anterior-posterior time to stabilization between the two legs. MLTTS-Asy: asymmetry in medial-lateral time to stabilization between the two legs. VTTS-Asy: asymmetry in vertical time to stabilization between the two legs. RVTTS-Asy: asymmetry in resultant vector time to stabilization between the two legs. Bonferroni adjustment was not made [39, 40].

**Fig 1:**
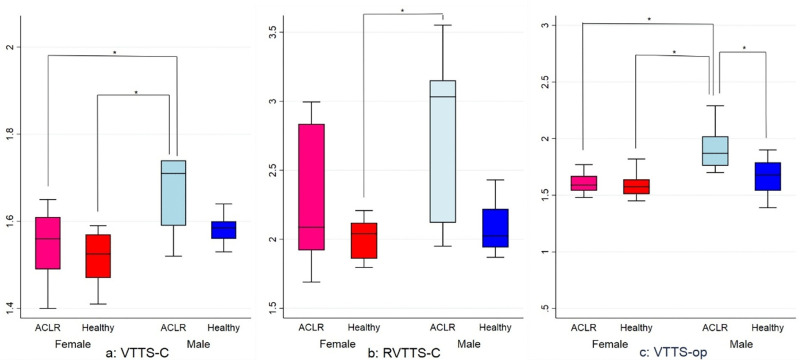
Pair-wise comparison results for TTS variables (in seconds); a:VTTS-C, b:RVTTS-C and c:VTTS-op. * Significant difference between sub-groups‌‌.

The Kruskal Wallis test revealed significant differences between the subgroups (female ACLR, female healthy, male ACLR and male healthy) for the combined vertical time to stabilization VTTS-C (p = 0.01), RVTTS-C (p = 0.02), and VTTS-op (p < 0.01). (see Table 4) Subsequent pairwise Dunn’s tests, with Bonferroni adjustment, demonstrated that males in the ACLR group exhibited significantly higher VTTS-C compared to females in the ACLR group (p = 0.03, δ = −0.69; 95% CI [−0.89, −0.26]) and females in the control group (p < 0.01, δ = −0.74; 95% CI [−0.93, −0.25]) (see Fig 1a). Similarly, males with ACLR displayed elevated RVTTS-C compared to healthy females (p = 0.01, δ = −0.73; 95% CI [−0.91, −0.31]) (see Fig 1b). Pairwise analysis on VTTS-op showed that males with ACLR exhibited significantly higher values than females with ACLR (p < 0.01, δ = −0.82; 95% CI [−0.96, −0.39]) and healthy females (p < 0.01, δ = −0.92; 95% CI [−0.98, −0.64]), as well as compared to healthy males (p = 0.03, δ = −0.71; 95% CI [−0.90, −0.28]) (see Fig 1c). No other pairwise comparisons reached statistical significance.

**Table 4:** Subgroup differences- TTS outcomes.

OUTCOMES	ACLR		Healthy		df	*X* ^*2*^	p-value
	Female (n = 10)	Male (n = 11)	Female (n = 10)	Male (n = 10)			
APTTS-C	1.93 (0.13)	2.12 (0.40)	2.04 (0.27)	2.05 (0.23)	3	3.78	0.29
MLTTS-C	2.33 (0.27)	2.27 (0.35)	2.35 (0.66)	2.09 (0.31)	3	4.72	0.19
VTTS-C	1.56 (0.12)	1.71 (0.15)	1.53 (0.10)	1.56 (0.14)	3	12.76	0.01*
RVTTS-C	2.09 (0.91)	3.03 (1.03)	2.04 (0.52)	2.02 (0.27)	3	9.87	0.02*
APTTS-op	2.13 (0.42)	1.97 (0.70)	2.15 (0.21)	2.22 (0.40)	3	3.05	0.38
MLTTS-op	1.86 (0.31)	2.12 (0.47)	1.83 (0.45)	1.81 (0.60)	3	3.07	0.38
VTTS-op	1.59 (0.13)	1.87 (0.26)	1.58 (0.13)	1.68 (0.25)	3	16.94	<0.01*
RVTTS-op	2.76 (0.33)	2.87 (0.73)	2.85 (0.23)	2.87 (0.51)	3	0.01	1.00
APTTS-nop	2.02 (0.52)	2.19 (0.16)	2.18 (0.28)	2.36 (0.61)	3	1.20	0.75
MLTTS-nop	1.80 (0.55)	2.03 (0.38)	1.86 (0.24)	1.80 (0.62)	3	3.94	0.27
VTTS-nop	1.59 (0.15)	1.72 (0.11)	1.59 (0.11)	1.66 (0.15)	3	5.99	0.11
RVTTS-nop	2.93 (0.37)	2.99 (0.45)	2.92 (0.21)	2.98 (0.85)	3	1.87	0.60
APTTS-Asy	0.03 (0.19)	−0.01 (0.44)	0.01 (0.12)	0.01 (0.44)	3	0.62	0.89
MLTTS-Asy	0.08 (0.23)	0.05 (0.27)	0.06 (0.26)	−0.01 (0.13)	3	0.43	0.93
VTTS-Asy	0.03 (0.16)	0.05 (0.40)	0.06 (0.18)	0.07 (0.23)	3	0.08	0.99
RVTTS-Asy	0.05 (0.37)	−0.07 (0.53)	0.01 (0.10)	0.01 (0.37)	3	0.79	0.89

Data reported in median and inter-quartile range (IQR). APTTS-C: anterior-posterior time to stabilization combined from both force plates. MLTTS-C: medial-lateral time to stabilization combined from both force plates. VTTS-C: vertical time to stabilization combined from both force plates. RVTTS-C: resultant vector time to stabilization combined from both force plates. APTTS-op: anterior-posterior time to stabilization on the operated leg. MLTTS-op: medial-lateral time to stabilization on the operated leg. VTTS-op: vertical time to stabilization on the operated leg. RVTTS-op: resultant vector time to stabilization on the operated leg. APTTS-nop: anterior-posterior time to stabilization on the non-operated leg. MLTTS-nop: medial-lateral time to stabilization on the non-operated leg. VTTS-nop: vertical time to stabilization on the non-operated leg. RVTTS-nop: resultant vector time to stabilization on the non-operated leg. APTTS-Asy: asymmetry in anterior-posterior time to stabilization between the two legs. MLTTS-Asy: asymmetry in medial-lateral time to stabilization between the two legs. VTTS-Asy: asymmetry in vertical time to stabilization between the two legs. RVTTS-Asy: asymmetry in resultant vector time to stabilization between the two legs. Bonferroni adjustment was not made [39, 40].

### Postural stability indices

**Fig 2:**
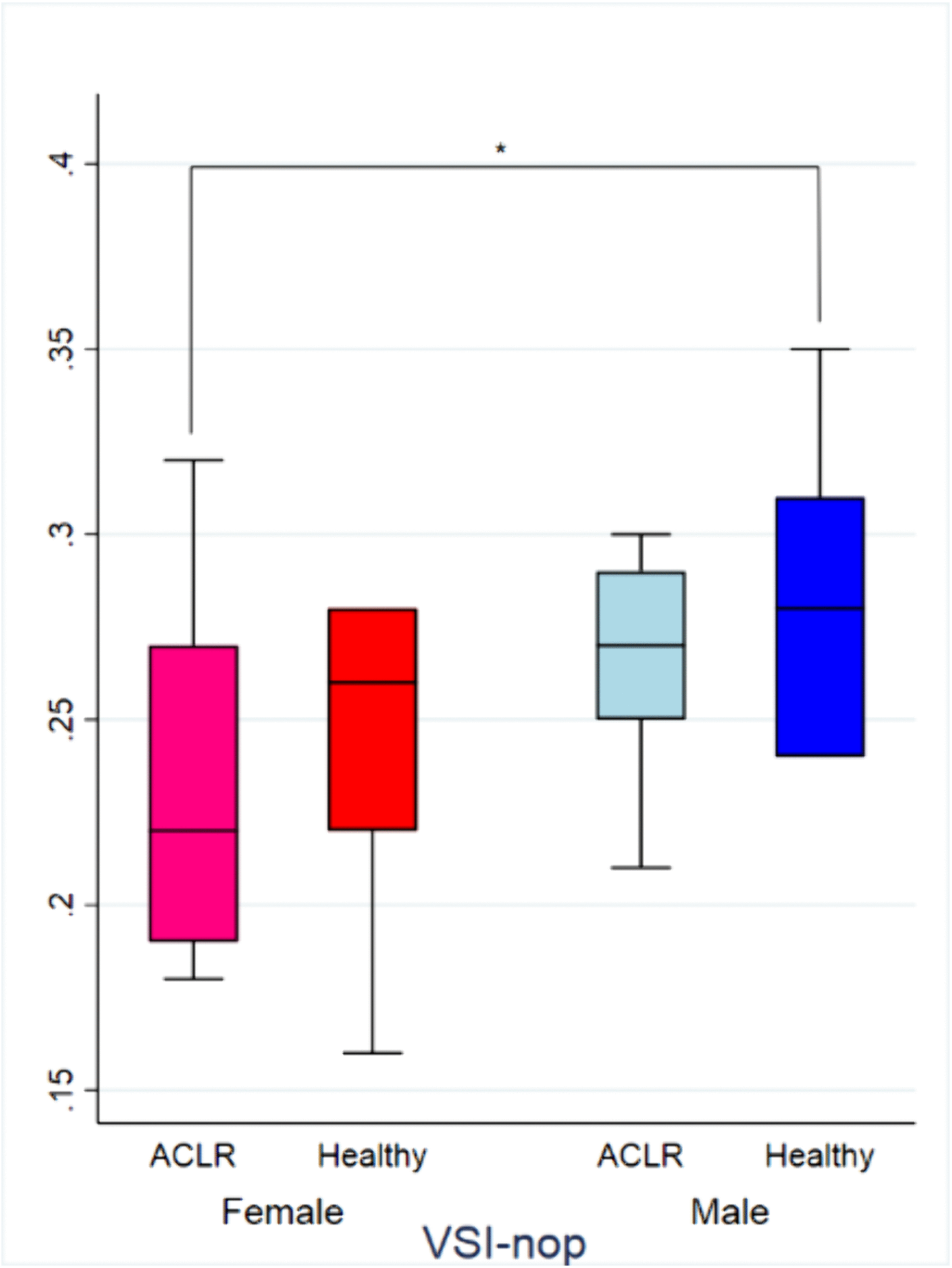
Pair-wise comparison results for the VSI-nop. * Significant difference between sub-groups.

No differences were found between the ACLR and control groups in any of the PSI variables. (see Table A in S2 File). However, when examining sex and ACLR status, we identified a significant difference between the four subgroups for the VSI-nop with a p = 0.05. (see Table B in S2 File) Pair wise analysis revealed that female participants with ACLR demonstrated higher VSI-nop values when compared to healthy males only (p = 0.04, δ = −0.63; 95% CI [−0.88, −0.11]). (see Fig 2) No interaction between sex and other PSI variables were identified.

## Discussion

Our study evaluated the differences in TTS and PSIs outcomes between individuals post-ACLR and healthy controls, as well as exploring sex differences. We examined our participants’ dynamic postural stability after double-leg CMJ (DL-CMJ) landing which allowed us to better capture the complex biomechanical and neuromuscular strategies individuals follow after ACLR [18]. Our results demonstrated that individuals with ACLR took a longer time to stabilize as was indicated by the significantly higher RVTTS-C and VTTS-op values when compared to healthy controls. This longer TTS may reflect persistent neuromuscular deficit and altered motor control strategies following ACLR, which may compromise the ability to rapidly achieve postural stability following landing. Future studies should investigate the underlying mechanisms contributing to these deficits. The findings also showed significant differences between sex and the ACLR status in the combined vertical time to stabilization (VTTS-C), RVTTS-C, VTTS-op and VSI-nop. Specifically, males post-ACLR demonstrated higher values in VTTS-C and VTTS-op when compared to females with ACLR, and higher VTTS-op compared to healthy males. The observed increase in TTS suggests that dynamic postural stability remains compromised following ACLR, particularly during tasks requiring rapid stabilization. This may reflect incomplete restoration of sensorimotor function despite clinical recovery.

While these findings were interpreted in the context of neuromuscular control, surgical factors such as graft placement and tunnel orientation may also influence dynamic knee stability. Previous studies have shown that variations in femoral tunnel orientation can affect knee loading patterns during landing tasks [41]. As detailed surgical parameters were not available in the present study, the observed differences in TTS may reflect a combination of neuromuscular and mechanical influences.

Similar to our findings, previous studies demonstrated higher TTS outcomes among individuals post-ACLR [20,21,42]. Patterson and Delahunt (2013) compared APTTS, MLTTS and RVTTS between female participants with and without ACLR while performing single leg forward landing and single-leg diagonal landing. They reported higher values in all parameters among the ACLR group with diagonal landing but not with forward landing [42]. Webster et al (2010) reported an increased RVTTS of 0.11 seconds among elite female athlete with ACLR when compared to a matching control group while performing a single leg drop jump [21]. However, more recent studies report contradictory findings. Aghdam et al (2024) studied VTTS in a group of males (15 with ACLR, and 15 control) while performing single-leg drop jump landing, and found no differences between the two groups [43]. However, all the ACLR participants in that study had already returned to sports and were at least two years post-surgery, so it is possible that our results may only be applicable to the recovery period. Similarly, Chaput et al (2022) reported no difference in the RVTTS between the ACLR group (n = 16, female = 10) and the control group (n = 15, female = 9) when they performed forward jump landing on a single leg [44]. The conflicting results in the previous studies could be due to the timing of the jumps relative to their surgery, heterogeneity in jump types, population characteristics, TTS outcomes being studied as well as the methods of calculations [34]. Our study found no differences in the RVTTS-op and the RVTTS-nop between the ACLR and the corresponding limbs in the control group. However, the RVTTS-C was significantly higher in the ACLR group (p = 0.03) with a moderate effect size δ = 0.40, 95% CI (0.04, 0.67). This finding suggest that the RVTTS-C is more sensitive in detecting between-group differences and highlights the complexity of postural stability, particularly after DL-CMJ. This may be explained by the fact that double-leg landing is an inherently bilateral tasks requiring inter-limb control, where compensatory strategies in one limb may mask deficits when limbs are analyzed separately. In contrast, combined TTS measures may better capture the neuromuscular control required to stabilize the body following landing. This may also indicate that individuals post-ACLR depend on compensatory weight-bearing strategies between limbs, which may mask unilateral deficit when assessed separately but become evident when evaluating whole-body stability [45]. Therefore, the inclusion of TTS-C measures may enhance the sensitivity for residual functional deficits following ACLR.

Furthermore, our study demonstrated significant differences in sex and ACLR status in specific TTS outcomes: VTTS-C, RVTTS-C and VTTS-op. No previous studies have examined the impact of sex and ACLR status on postural stability. However, given that secondary ACL injuries are more common in females [46–48] and that deficits in postural stability, measured on a balance platform, can predict a secondary ACL injury [49], one would expect higher TTS values among females. Contrary to these expectations, our findings suggest that males post-ACLR exhibited higher TTS values across various measures compared to females in both the ACLR and control groups. One possible explanation is that males may adopt different landing strategies, potentially relying more on stiffness [50], which could prolong stabilization time. Additionally, jump height was not controlled in this study, and differences in participants in different sex groups may have influenced landing mechanisms, and accordingly, stabilization time. Higher jump heights may result in greater GRFs and longer TTS. Alternatively, differences in neuromuscular control, strength, or movement variability between sexes may also contribute to these findings. This challenges the notion that reduced postural stability is a risk factor for secondary ACL injuries and highlights postural stability as a complex phenomenon that requires further sex-specific investigations, particularly in the ACLR population.

Interestingly, no significant differences were observed between the ACLR and healthy control groups across PSI variables. Our findings agree with previous literature studying PSI in the ACLR population. Robey et al (2021) reported no significant differences in DPSI and no association between sex and ACLR status during a jump landing task [51]. Similarly, Head et al (2019) compared DPSI between 15 individuals with ACLR and 15 controls and reported no differences while performing forward jump-landing, lateral jump-landing and diagonal jump-landing [52]. Our study identified a significant difference for the VSI-nop between sex and ACLR status with the pairwise analysis identifying that the difference occurred between female ACLR and healthy males.

This study is not without limitations. The cross-sectional design of the study prevents causal inferences. Our first objective was to comprehensively explore differences between people with and without ACLR comparing a total of 32 parameters. We didn’t employ Bonferroni adjustment for those comparison, which may have increased the risk of type 1 error. However, we did imbed the Bonferroni adjustment for all pairwise comparisons. Furthermore, we did not control for other covariates that could have influenced our results, such as concomitant meniscus injuries or graft type, due to the small sample size. Additionally, between group differences in differences in trunk fat percentage, were not controlled for and may have influenced stabilization demands during landing. Variations in mass distribution could affect the mechanical load and eccentric control required to stabilize the center of mass, potentially contributing to differences in TTS outcomes [53].

In addition, the subgroup analysis examining sex differences were conducted on smaller sample sizes and may have been underpowered; therefore, these findings should be interpreted with caution and considered exploratory in nature. Additionally, limb dominance was not accounted for and may influence lower-limb biomechanics and stability outcomes following ACLR [54]. Therefore, differences observed in the operated limb may be affected by whether the injury occurred in the dominant or non-dominant limb and should be interpreted with caution.

Moreover, although participants were instructed to perform the countermovement jump to approximately 90° of knee flexion, this depth was not instrumentally controlled, which may have introduced variability in movement execution across participants. However, this approach was chosen to preserve ecological validity, as it better reflects natural movement patterns during functional tasks. Given that the same instructions and supervision were applied consistently among participants, such variability is unlikely to influence between-group comparisons.

GRF data were processed using a 100-millisecond window to reduce noise. While this approach improves signal stability and the reliability of detecting post-landing stabilization, it may reduce a minimal temporal smoothing effect that could slightly influence the estimation of TTS. However, the same data processing procedure was applied consistently across all participants and trials, minimizing the likelihood of systematic bias between groups.

Additionally, participants with <80% quadriceps strength symmetry were excluded to ensure a functionally recovered cohort. While this reduces variability, it may introduce selection bias and limit generalizability to individuals with greater strength deficits, in whom impairments in postural stability may be more pronounced.

Finally, the findings may not be generalizable beyond young, physically active individuals, and the relatively small sample size may limit statistical power. This study focused on force plate–derived kinetic measures and did not include kinematic, joint moment, center of pressure analysis, EMG, or proprioceptive assessments, which could provide further insight and should be considered in future research.

With the observed sex differences in TTS outcomes, further research is needed to elucidate the underlying mechanisms contributing to differences between males and females. Longitudinal studies examining the changes of postural stability post-ACLR compared to healthy controls could provide valuable insights into their impact on long-term functional outcomes. Additionally, exploring the role of psychological factors, such as fear of re-injury, could further refine our understanding of recovery and return-to-sport decisions.

## Conclusion

Our findings demonstrate that individuals post-ACLR exhibit prolonged time to stabilization (TTS) during a functional double-leg landing task compared to healthy controls, indicating persistent deficits in dynamic postural control despite clinical recovery. The presence of sex-specific differences further highlights the complexity of neuromuscular adaptations following ACLR.

Importantly, the lack of differences in postural stability indices (PSIs), alongside significant differences in TTS measures, suggests that TTS may be a more sensitive metric for detecting residual functional deficits during dynamic tasks. These findings emphasize the importance of incorporating time-based dynamic stability measures, particularly during bilateral functional movements, into clinical assessment.

From a clinical perspective, evaluating dynamic postural stability through measures such as TTS may enhance return-to-sport decision by identifying deficits that are not captured through traditional strength or static balance assessments. This may help clinicians better detect incomplete neuromuscular recovery and reduce the risk of re-injury.

Future research should further investigate the mechanisms underlying sex differences and explore how dynamic stability measures can be integrated into rehabilitation protocols to optimize recovery post-ACLR.

## Supporting information

S1 File Dataset supporting the findings of this study. (XLSX)

S2 File Supporting tables. A Table. Comparisons of PSI outcomes. B Table. Subgroups differences- PSI outcomes.
(DOCX)

S1 Text Inclusivity in global research questionnaire. (DOCX)
