# Supplementary material for: Dynamic postural stability in individuals with ACL reconstruction versus healthy controls with insights into sex differences: A cross-sectional study
Source: PLoS One. 2026 Jun 12;21(6):e0351496. doi: 10.1371/journal.pone.0351496 (PMC13262813; doi:10.1371/journal.pone.0351496)
Supplement: S2 File — A Table. Comparisons of PSI outcomes. B Table. Subgroups differences- PSI outcomes. (DOCX) [file pone.0351496.s002.docx]

# Supporting file 2. PSI outcomes

Table A - Comparisons of PSI outcomes

| OUTCOMES | ACLR (n = 21) | Healthy (n = 20) | P-value |
| --- | --- | --- | --- |
| APSI-C | 0.69 (0.16) | 0.71 (0.22) | 0.86 |
| MLSI-C | 0.63 (0.24) | 0.64 (0.21) | 0.25 |
| VSI-C | 0.24 (0.04) | 0.25 (0.04) | 0.29 |
| DPSI-C | 0.94 (0.20) | 1.06 (0.20) | 0.25 |
| APSI-op | 0.60 (0.17) | 0.68 (0.25) | 0.45 |
| MLSI-op | 0.61 (0.46) | 0.62 (0.37) | 0.60 |
| VSI-op | 0.24 (0.06) | 0.25 (0.04) | 0.21 |
| DPSI-op | 0.89 (0.39) | 0.99 (0.30) | 0.40 |
| APSI-nop | 0.72 (0.28) | 0.70 (0.18) | 0.67 |
| MLSI-nop | 0.58 (0.40) | 0.68 (0.45) | 0.35 |
| VSI-nop | 0.27 (0.06) | 0.26 (0.05) | 0.44 |
| DPSI-nop | 1.01 (0.39) | 1.07 (0.31) | 1.00 |
| APSI-Asy | 0.05 (0.25) | 0.02 (0.18) | 0.71 |
| MLSI-Asy | 0.48 (0.48) | 0.37 (0.30) | 0.77 |
| VSI-Asy | 0.01 (0.10) | -0.02 (0.09) | 0.30 |
| DPSI-Asy | 0.41 (0.46) | 0.21 (0.29) | 0.77 |
| Data reported in median and inter-quartile range (IQR). APSI-C: anterior-posterior stability index combined from both force plates. MLSI-C: medial-lateral stability index combined from both force plates. VSI-C: vertical stability index combined from both force plates. DPSI-C: dynamic postural stability index combined from both force plates. APSI-op: anterior-posterior stability index on the operated leg. MLSI-op: medial-lateral stability index on the operated leg. VSI-op: vertical stability index on the operated leg. DPSI-op: dynamic postural stability index on the operated leg. APSI-nop: anterior-posterior stability index on the non-operated leg. MLSI-nop: medial-lateral stability index on the non-operated leg. VSI-nop: vertical stability index on the non-operated leg. DPSI-nop: dynamic postural stability index on the non-operated leg. APSI-Asy: asymmetry in anterior-posterior stability index between the two legs. MLSI-Asy: asymmetry in medial-lateral stability index between the two legs. VSI-Asy: asymmetry in vertical stability index between the two legs. DPSI-Asy: asymmetry in dynamic postural stability index between the two legs. Bonferroni adjustment was not made [33,34] | | | |

Table B- Subgroups differences- PSI outcomes

| OUTCOMES | ACLR | | Healthy | | df | F or *X^2^* | P-value |
| --- | --- | --- | --- | --- | --- | --- | --- |
|  | **Female (n=10)** | **Male (n=11)** | **Female (n=10)** | **Male (n=10)** |  |  |  |
| APSI-C | 0.70 (0.13) | 0.69 (0.28) | 0.66 (0.22) | 0.77 (0.30) | 3 | 2.37 | 0.50 |
| MLSI-C | 0.66 (0.24) | 0.58 (0.19) | 0.66 (0.16) | 0.63 (0.30) | 3 | 2.36 | 0.50 |
| VSI-C | 0.23 (0.05) | 0.24 (0.04) | 0.24 (0.03) | 0.26 (0.05) | 3 | 4.32 | 0.23 |
| DPSI-C | 0.99 (0.14) | 0.91 0.17) | 1.00 (0.10) | 1.13 (0.31) | 3 | 3.08 | 0.38 |
| APSI-op | 0.59 (0.23) | 0.63 (0.21) | 0.63 (0.26) | 0.69 (0.39) | 3 | 2.59 | 0.46 |
| MLSI-op | 0.63 (0.46) | 0.58 (0.73) | 0.80 (0.35) | 0.51 (0.40) | 3 | 2.28 | 0.52 |
| VSI-op | 0.24 (0.08) | 0.24 (0.05) | 0.26 (0.03) | 0.25 (0.04) | 3 | 1.67 | 0.64 |
| DPSI-op | 0.99 (0.39) | 0.84 (0.71) | 1.02 (0.26) | 0.96 (0.27) | 3 | 0.81 | 0.98 |
| APSI-nop | 0.75 (0.19) | 0.69 (0.37) | 0.66 (0.20) | 0.74 (0.20) | 3 | 2.93 | 0.40 |
| MLSI-nop | 0.61 (0.36) | 0.58 (0.16) | 0.68 (0.23) | 0.86 (0.40) | 3 | 2.35 | 0.50 |
| VSI-nop | 0.22 (0.08) | 0.27 (0.04) | 0.26 (0.06) | 0.28 (0.07) | 3 | 7.82 | **0.05*** |
| DPSI-nop | 1.08 (0.35) | 1.01 (0.43) | 0.95 (0.25) | 1.18 (0.28) | 3 | 3.39 | 0.34 |
| APSI-Asy | 0.16 (0.42) | 0.00 (0.30) | 0.05 (0.11) | -0.03 (0.20) | 3 | 0.79 | 0.85 |
| MLSI-Asy | 0.49 (0.28) | 0.41 (0.72) | 0.37 (0.29) | 0.46 (0.33) | 1 | 2.31 | 0.51 |
| VSI-Asy | 0.05 (0.09) | 0.00 (0.11) | -0.02 (0.07) | -0.02 (0.09) | 1 | 2.43 | 0.49 |
| DPSI-Asy | 0.39 (0.39) | 0.41 (0.49) | 0.24 (0.21) | 0.18 (0.38) | 3 | 0.24 | 0.97 |
| Data reported in median and inter-quartile range (IQR). APSI-C: anterior-posterior stability index combined from both force plates. MLSI-C: medial-lateral stability index combined from both force plates. VSI-C: vertical stability index combined from both force plates. DPSI-C: dynamic postural stability index combined from both force plates. APSI-op: anterior-posterior stability index on the operated leg. MLSI-op: medial-lateral stability index on the operated leg. VSI-op: vertical stability index on the operated leg. DPSI-op: dynamic postural stability index on the operated leg. APSI-nop: anterior-posterior stability index on the non-operated leg. MLSI-nop: medial-lateral stability index on the non-operated leg. VSI-nop: vertical stability index on the non-operated leg. DPSI-nop: dynamic postural stability index on the non-operated leg. APSI-Asy: asymmetry in anterior-posterior stability index between the two legs. MLSI-Asy: asymmetry in medial-lateral stability index between the two legs. VSI-Asy: asymmetry in vertical stability index between the two legs. DPSI-Asy: asymmetry in dynamic postural stability index between the two legs. Bonferroni adjustment was not made [33,34] | | | | | | | |
